# Clinicopathologic Features of Gastric Schwannoma: 8-Year Experience at a Single Institution in China

**DOI:** 10.1097/MD.0000000000001970

**Published:** 2015-11-13

**Authors:** Kaixiong Tao, Weilong Chang, Ende Zhao, Rui Deng, Jinbo Gao, Kailin Cai, Guobin Wang, Peng Zhang

**Affiliations:** From the Department of Gastrointestinal Surgery, Union Hospital, Tongji Medical College, Huazhong University of Science and Technology, Wuhan, Hubei, China.

## Abstract

To explore the clinicopathologic characteristics, diagnosis, treatment, and prognosis of gastric schwannoma in the imatinib era.

The clinicopathologic characteristics and postoperative outcomes of patients diagnosed with gastric schwannoma at our institution between January 2007 and February 2015 were retrospectively collected and analyzed.

The main patient complaint was epigastric pain or discomfort. Tumor sizes ranged from 15 to 80 mm (mean, 57.1 mm). In 17 patients, the tumors were located in the body of the stomach. A total of 20 patients were preoperatively misdiagnosed with a gastrointestinal stromal tumor. The rate of correct preoperative diagnosis was only 3.3%. All patients underwent surgical resection and showed strong S-100 protein positivity. Laparoscopic surgery for gastric schwannoma was associated with less blood loss and a shorter postoperative hospital stay than open surgery (*P* < 0.01). Total 28 patients were disease free without recurrence or metastasis at a median follow-up time of 50 months.

Gastric schwannoma is often preoperatively misdiagnosed as gastric gastrointestinal stromal tumor. Laparoscopic resection of gastric schwannoma is considered safe and effective, and it may be the preferred surgery for most small- and moderate-sized tumors. The long-term outcome is excellent, as this type of neoplasm is uniformly benign.

## INTRODUCTION

According to the World Health Organization, gastric tumors are classified into 2 large categories, epithelial and nonepithelial, based on the cell origin.^1^ Epithelial neoplasms originate from the mucosa, whereas nonepithelial neoplasms arise from deep within the mucosa and are also termed mesenchymal tumors of the gastric wall.^2^ In contrast with epithelial tumors, mesenchymal tumors arise from submucosa, muscularis propria, or serosa, and they are often well circumscribed, with an intact overlying mucosa.^3^ Furthermore, mesenchymal tumors are rather rare, comprising 0.1% to 3% of all gastrointestinal tumors,^4^ and they consist of a spectrum of spindle cell tumors, mainly including gastrointestinal stromal tumor (GIST), leiomyoma or leiomyosarcoma, and schwannoma.^5^ Among these tumors, GIST represents approximately 80% of mesenchymal tumors of the gastrointestinal tract, with the stomach being the most common primary site. Schwannoma rarely occurs in the gastrointestinal tract, representing only 3% of all gastrointestinal mesenchymal tumors.^6^ As with GIST, the most common primary site of schwannoma is also the stomach. It has been reported that gastric schwannoma represents 0.2% of all gastric tumors.^7,8^

Previously, schwannoma and GIST were often misdiagnosed as leiomyoma or leiomyosarcoma.^7,9^ Only in the last decade, with new developments in immunohistochemistry, have schwannoma and GIST emerged as separate entities. The diagnosis of schwannoma is based on positive immunohistochemical staining for S-100 protein and negative results for CD117, CD34, desmin, and smooth muscle actin (SMA), whereas GIST is typically positive for CD117, DOG-1, and CD34 but negative for S-100 protein. It, however, is difficult to distinguish between gastric schwannoma and gastric GIST before surgery.

To date, few reports are available describing gastric schwannoma in the literature. In addition, in most previous studies, the diagnosed cases were scattered across a wide time range. Large single-institution series of patients with gastric schwannoma diagnosed within the past 10 years have been lacking. Thus, we analyzed 30 patients with gastric schwannoma for whom diagnosis was confirmed after January 2007. The purpose of this study was to gain a better understanding of gastric schwannoma. To the best of our knowledge, our series is the largest single-institution review of gastric schwannoma in the imatinib era.

## METHODS

Thirty patients with gastric schwannoma treated at Wuhan Union Hospital between January 2007 and February 2015 were recruited for this study. The admission criterion was schwannoma of the stomach confirmed by pathologic examination at our institution. Patient data, including patient demographics, clinical presentation, preoperative imaging evaluation results, operative time, intraoperative blood loss amount, histopathology, postoperative complications, length of postoperative hospital stay, and follow-up results, were retrospectively analyzed. All patients provided written informed consent. The institutional review board and the ethics committee of Union Hospital deemed that an ethical review was not required for this retrospective analysis.

### Statistical Analysis

Statistical analysis was performed using SPSS version 13.0 software (SPSS Inc., Chicago, IL). Significant differences were evaluated using Fisher exact test for categorical data and Student's *t*-test for quantitative data. A *P* < 0.05 was considered to indicate a statistically significant difference.

## RESULTS

### Clinical Findings

The clinical data for the 30 patients (11 men and 19 women) are summarized in Table 1. The patients’ ages ranged from 38 to 79 years (mean age, 56.9 years; median age, 57 years). The main complaint was epigastric discomfort or pain, which was reported by 14 patients (46.7%). Nine patients (30%) were asymptomatic, and their tumors were detected incidentally by routine physical examination. Three patients (10%) presented with gastrointestinal bleeding, including melena (n = 3) and hematemesis (n = 1). In 2 patients, the tumors were incidentally discovered during surgery or examination for concomitant diseases. Other complaints included poor appetite (n = 1) and anemia because of chronic blood loss (n = 1).

**TABLE 1:**
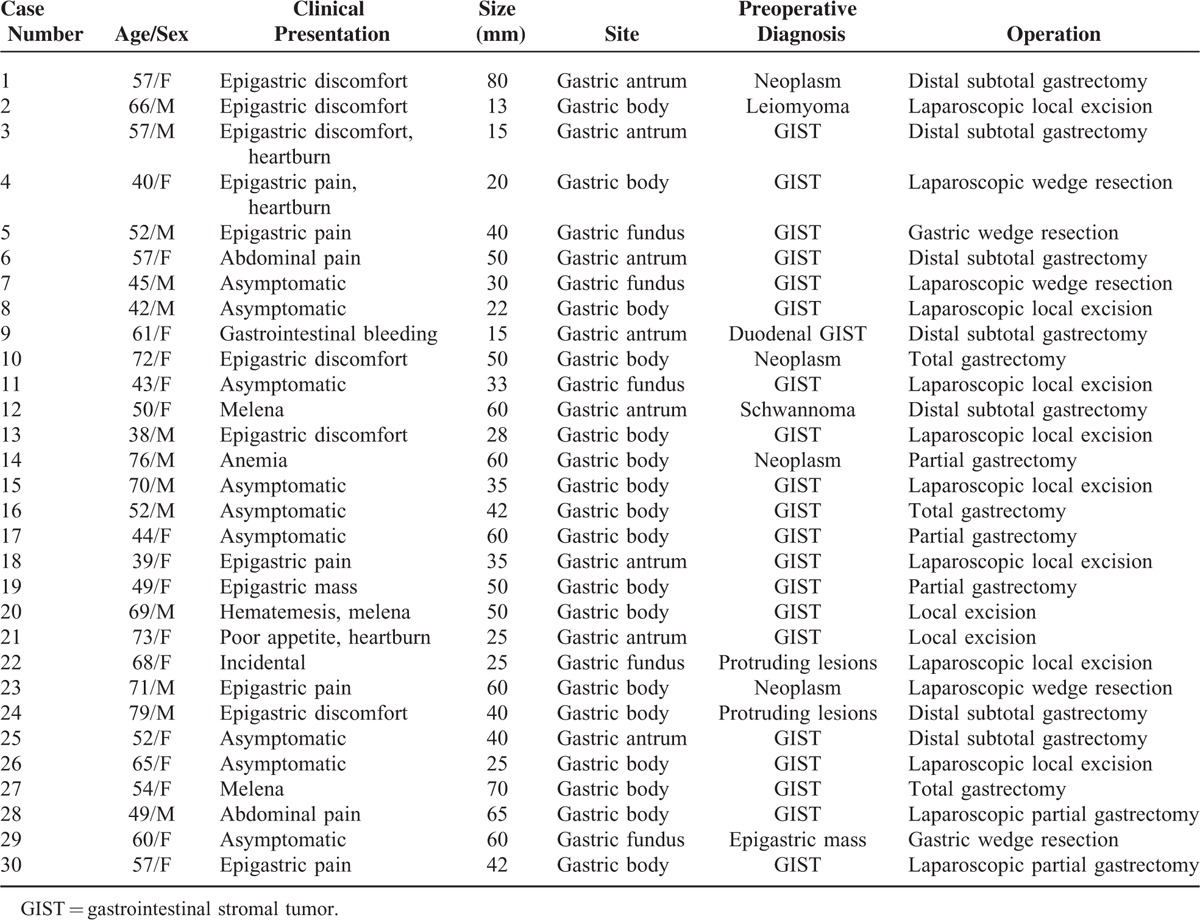
Clinicopathologic Characteristics of 30 Cases of Gastric Schwannoma

Five patients had concomitant diseases, including cholelithiasis (n = 2), hepatic cyst, hepatic cavernous hemangioma, and duodenal GIST. None of the patients had a history of neurofibromatosis type 1 or type 2 syndrome.

### Preoperative Evaluation

Endoscopy was performed on 22 patients at our hospital, with submucosal mass as the main finding. Mucosal ulceration was observed in 3 patients (13.6%). Eighteen patients underwent endoscopic ultrasonography, which demonstrated hypoechoic, submucosal masses arising from the fourth proper muscle layer. Furthermore, endoscopic biopsy was performed on 3 patients, but all were found to have normal mucosa. Another 2 patients underwent endoscopic ultrasound-guided fine-needle aspiration (EUS-FNA) biopsy, and 1 was preoperatively diagnosed with gastric schwannoma.

Computed tomography (CT) was performed for 16 patients, of whom 12 (75%) showed a homogeneous enhancement pattern. The tumor growth patterns were diverse and included endoluminal (n = 5), exogastric (n = 8), and intramural (n = 3) growth. High accumulation (maximal standardized uptake value, 6.43) coincident with the tumor was found in 1 patient by 18F-fluorodeoxyglucose-positron emission tomography (FDG-PET). Tumor markers were examined in 13 patients, and all were negative. Among these patients, the preoperative diagnoses were gastric GIST (n = 20), gastric neoplasm (n = 4), gastric protruding lesion (n = 2), gastric schwannoma (n = 1), gastric leiomyoma (n = 1), left epigastric mass (n = 1), and duodenal GIST (n = 1). Only 3.3% of the preoperative diagnoses matched the pathologic diagnoses.

### Pathologic and Histologic Findings

**FIGURE 1:**
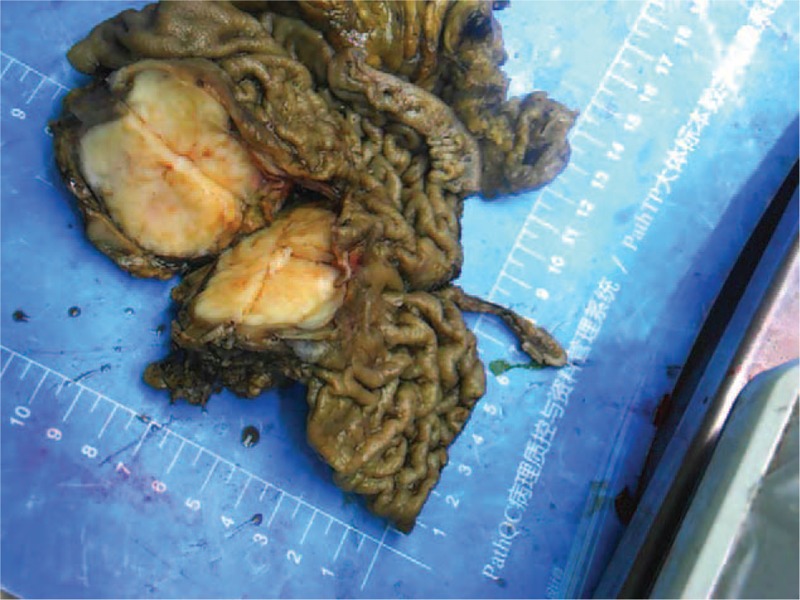
Gross appearance of gastric schwannoma. The tumor is homogeneous, firm, and gray-white, without ulceration, necrosis, or hemorrhage.

**FIGURE 2:**
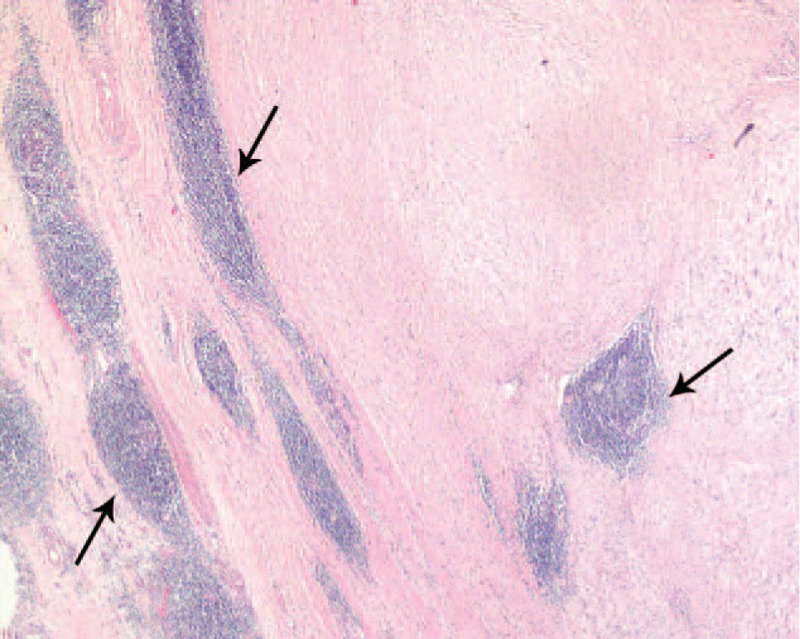
Tumor cells are surrounded by a characteristic peripheral lymphoid cuff (*arrows*). The image was captured under 20× magnification.

The tumors tended to be located in the middle portion of the stomach, including the fundus, body, and antrum, in 5, 17, and 8 patients, respectively. The maximal diameters of the tumors ranged from 13 to 80 mm (mean, 41.3 mm). Ulceration of the mucosa was observed in 4 patients (13.3%). No necrosis, cystic changes, or calcification were detected in any of these tumors. Gross examination showed that the gastric schwannomas were homogeneous and firm and that the colors of the cut surfaces were yellow, yellowish, or gray-white (Figure 1). In all patients, the tumors were composed of spindle cells that were arranged mainly in small bundles or in a woven pattern, as observed by microscopic examination. Tumor cells were surrounded by a peripheral lymphoid cuff in 26 patients (86.7%), and germinal center formation was detected in 19 patients (63.3%) (Figure 2).

### Immunohistochemical Findings and Genetic Studies

**FIGURE 3:**
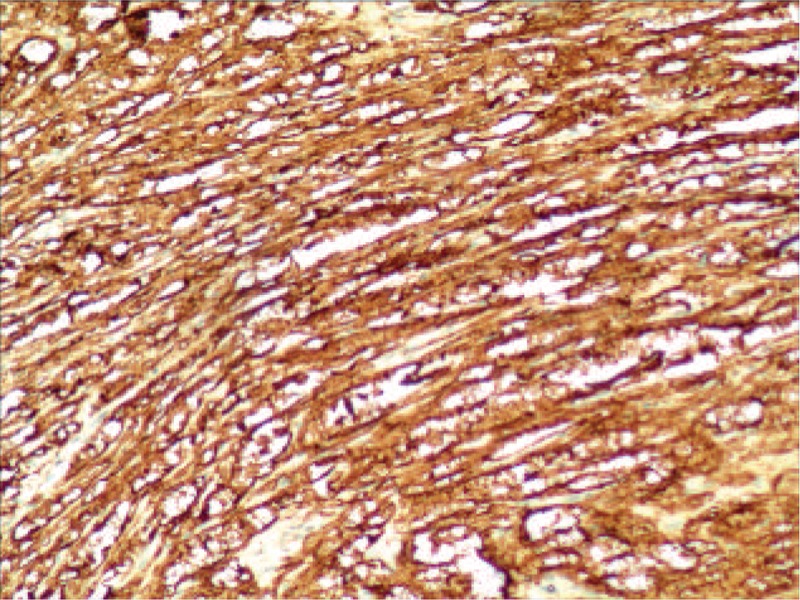
Immunohistochemistry shows diffuse strong positive staining for S-100 protein. The image was captured under 100× magnification.

Immunohistochemical staining revealed strong S-100 protein positivity in all 30 of the examined patients (Figure 3). Twenty-seven patients were negative for CD34, whereas only 3 showed focal CD34 expression. All examined patients were negative for CD117, DOG-1, SMA, and desmin. The Ki-67 index was less than 2% in all patients, indicating a low proliferation rate. Mutational analysis was conducted for 1 patient (No. 9), in which KIT exons 9, 11, 13, and 17, and PDGFRA (platelet-derived growth factor receptor, alpha polypeptide) exons 12 and 18 were evaluated. A mutation in KIT exon 11 was found in the duodenal GIST case, whereas no mutation was detected in the gastric schwannoma case.

### Treatment

All patients underwent surgical treatment, including laparoscopic resection for 13 patients and open resection for 17. The surgical treatment included local excision or wedge resection (n = 11) and partial gastrectomy (n = 2) in the laparoscopic group, and local excision or wedge resection (n = 4), partial gastrectomy (n = 3), subtotal gastrectomy (n = 7), and total gastrectomy (n = 3) in the open surgery group. Four patients underwent additional procedures during treatment for gastric schwannoma. In the laparoscopic group, 1 patient underwent laparoscopic cholecystectomy, and 1 underwent left lateral hepatic lobectomy, and in the open surgery group, 1 patient received fenestration for a hepatic cyst, and 1 underwent cholecystectomy.

In our series, the median operating time was 135 minutes (range, 55–255 minutes), with a median estimated blood loss of 60 mL (range, 10–600 mL). In the open surgery group, 3 patients received blood transfusion during surgery. Postoperative surgical complications occurred in 2 patients in the open surgery group. Wound infection occurred in 1 of these patients, and early postoperative inflammatory small bowel obstruction occurred in the other. The median hospital stay was 10.5 days (range, 8–36 days). A comparison of the characteristics of the laparoscopic and open surgery groups is shown in Table 2. There was no significant difference between the laparoscopic and open surgery groups based on sex or age. The laparoscopic group, however, was superior to the open surgery group with respect to operation time (*P* = 0.006), estimated blood loss (*P* = 0.006), and postoperative hospital stay (*P* = 0.004).

**TABLE 2:**
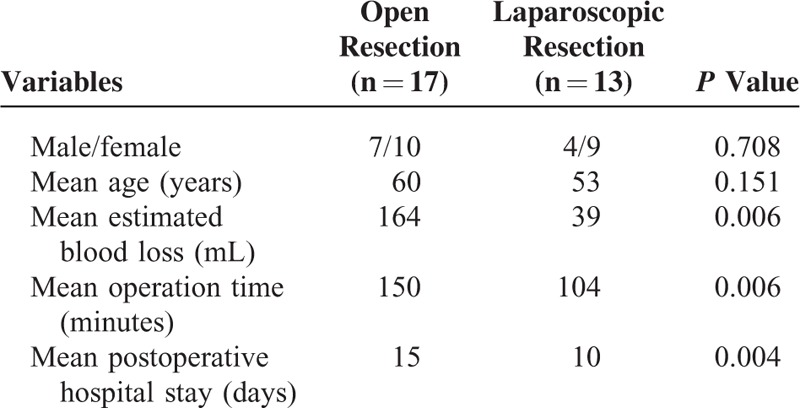
Comparisons of the Characteristics Between Laparoscopic and Open Resection of Gastric Schwannoma

### Follow-Up

Follow-up was completed for 28 patients (93.3%). All of these patients were disease free, without recurrence or metastases, at a median follow-up time of 50 months (range, 12–97 months).

## DISCUSSION

Schwannoma, also known as neurilemmoma or neurinoma, is a tumor originating from Schwann cells. Gastric schwannoma is a rare neoplasm that should be distinguished from other submucosal neoplasms of the stomach, such as GIST, leiomyoma or leiomyosarcoma, and lymphoma, some of which are malignant or have malignant potential. With recent advances in pathologic and immunohistochemical techniques, schwannoma and GIST have been recognized in the last 20 years as different primary gastrointestinal mesenchymal tumor entities. According to a recent classification, GIST accounts for approximately 80% of gastrointestinal mesenchymal tumors.^10^ Based on our data, gastric schwannoma is much less common than gastric GIST. At our institution, 267 patients were confirmed to have a gastric GIST during the same time period—that is, approximately 9 cases of gastric GIST were observed for each case of gastric schwannoma.

Gastric schwannoma occurs more frequently in patients ranging in age from 50 to 60 years and shows a greater prevalence in women^11^; in our series, the female:male ratio reached nearly 2:1. This type of tumor is usually slow growing and is located in the body of the stomach along the lesser curvature.^12,13^ Owing to this indolent growth pattern, gastric schwannoma is asymptomatic and in most cases is incidentally discovered during routine medical checkup.^8^ Fujiwara et al^14^ reported the identification of 13 patients (93%) via incidental findings upon cross-sectional imaging or endoscopy or during intraoperative visualization. In some cases, patients, however, may present with symptoms such as gastrointestinal bleeding, epigastric discomfort, or a palpable mass.^11,15^ In our study, approximately half of the patients presented with epigastric pain or discomfort; however, one-third of them were asymptomatic or were discovered incidentally, a finding that was not in accordance with those of previous studies.

Gastric schwannoma is usually detected preoperatively via endoscopy or cross-sectional imaging. Preoperative examination may be helpful for gaining the necessary information for preoperative diagnosis and for determining whether surgical resection is feasible. It, however, is very difficult to distinguish between gastric schwannoma and other types of gastric submucosal tumors by preoperative examination because of its rarity and lack of specific characteristics. On endoscopy, gastric schwannoma appears as an elevated submucosal mass that occasionally exhibits mucosal ulceration, making it indistinguishable from gastric GIST. Endoscopic ultrasonography scans can be used to delineate the full depth of a tumor and to direct needle biopsy. Zhong et al^16^ have noted that heterogeneous hypoechogenicity or isoechogenicity, a well-demarcated margin, fourth-layer origination, and a lack of cystic change may be considered as useful findings for the diagnosis of gastric schwannoma. Endoscopic ultrasound-guided fine-needle aspiration is an accurate method for the diagnosis of gastric submucosal tumors, and the diagnostic yield has been reported to be 43.3% to 52%.^17,18^ In our study, EUS-FNA was performed on 2 patients, but only 1 diagnosis was confirmed, as obtaining a sufficient amount of tissue was difficult. The National Comprehensive Cancer Network guidelines, however, do not recommend preoperative biopsy for primary resectable GIST, as there is a theoretical risk of tumor rupture and spread in association with poor prognosis.^19^ Therefore, considering the above information, preoperative biopsy is not routinely performed at our center.

Computed tomography is helpful for defining the exact location and extent of a tumor by revealing the displacement of the surrounding organs. On CT examination, gastric schwannoma exhibits homogeneous enhancement in most patients, and cystic changes are uncommon, consistent with our findings.^20,21^ Gastric GIST commonly, however, shows heterogeneous enhancement because of degenerative transformations, such as hemorrhage, necrosis, and cystic changes.^22^ 18F-fluorodeoxyglucose-positron emission tomography has been extensively used for the evaluation of various types of tumors, including GIST.^23,24^ Kamiyama et al^24^ have reported that fluorodeoxyglucose (FDG) uptake and the malignant potential of gastric GIST are strongly correlated. Increased FDG uptake, however, has also been reported in 3 patients of gastric schwannoma.^25,26^ The actual mechanism of high F-18 FDG uptake in gastric schwannoma has not yet been clarified in detail and may be related to intracellular glycolytic activity.^27^ Therefore, the value of FDG-PET as a preoperative diagnostic technique to differentiate gastric schwannoma from GIST is limited.

The definitive diagnosis of gastric schwannoma is determined by pathologic and immunohistochemical examination of surgical specimens. Schwannoma shows strong positive staining for S-100 protein and negative staining for CD117, CD34, desmin, and SMA.^6,13^ The S-100 staining pattern detected by immunohistochemistry is both nuclear and cytoplasmic. Gastric schwannoma may occasionally express CD34, but CD117, SMA, and desmin are uniformly negative. Macroscopically, gastric schwannoma has often been described as homogeneous, firm, or rubbery, and it rarely shows degenerative changes.^6^ Cystic changes, hemorrhage, and necrosis, however, are common in GIST.^28^ In the current study, no cystic change or necrosis was found, whereas ulceration was observed in only 4 gastric schwannoma patients. Microscopically, the tumors consisted of spindle cells with a prominent lymphoid cuff and were characterized by the absence of typical Verocay bodies, vascular hyalinization, and Antoni A and Antoni B areas.^29^ The genetic features of gastric schwannoma include a lack of KIT and PDGFRA mutations, in contrast with GIST.

In this study, the outcomes of the gastric schwannoma patients after surgical resection were excellent, with no recurrence, metastasis, or tumor-related mortality. Similarly, previous studies have indicated that gastric schwannoma is a benign neoplasm that is associated with an excellent prognosis.^6,7,13,30,31^ Malignant gastric schwannoma is extremely rare.^11^ In fact, previous diagnoses of malignant schwannoma predated the application of modern immunohistochemistry techniques; therefore, gastric schwannoma could not then be reliably distinguished from GIST. In our study, the preoperative diagnosis in most of the patients was gastric GIST. Owing to the uncertainty of the preoperative diagnosis, the treatment of choice for gastric schwannoma remains complete surgical resection, similar to the treatment for GIST. The type of operation to perform depends on the tumor location, size, and relationship with the surrounding organs. Currently, gastric GIST is viewed as a good indication for laparoscopic resection, regardless of tumor size.^32,33^ Our data showed that laparoscopic surgery for most small- and moderate-sized gastric schwannomas was associated with less blood loss and a shorter postoperative hospital stay compared with open surgery. Moreover, it is crucial to avoid intraoperative tumor rupture. In all patients, an incision protection sleeve was used during the operation, and the specimen was placed into a specimen retrieval bag to prevent tumor peritoneal seeding or wound seeding.

## CONCLUSIONS

Compared with gastric GIST, gastric schwannoma is a rarer gastric mesenchymal tumor with predominance in women in the imatinib era. This type of neoplasm is frequently located in the body of the stomach and predominantly occurs in older adults. Owing to their rare incidences and similar clinical manifestations, gastric schwannoma is typically misdiagnosed as gastric GIST before surgery. Complete margin-negative surgical resection is the curative treatment of choice. Laparoscopic resection of gastric schwannoma is considered safe and effective, and it may be the preferred resection technique for most patients. The long-term outcome is excellent, as this type of neoplasm is uniformly benign.
